# Smoothing the Undersampled Carpal Bone Model with Small Volume and Large Curvature: A Feasibility Study

**DOI:** 10.3390/life12050770

**Published:** 2022-05-23

**Authors:** Chengcheng Ji, Jianzhang Li, Maximilian Praster, Björn Rath, Frank Hildebrand, Jörg Eschweiler

**Affiliations:** 1 Department of Orthopaedics, Trauma and Reconstructive Surgery, RWTH Aachen University Hospital, 52074 Aachen, Germany; cji@ukaachen.de (C.J.); mpraster@ukaachen.de (M.P.); fhildebrand@ukaachen.de (F.H.); joeschweiler@ukaachen.de (J.E.); 2 Department of Orthopaedic Surgery, Klinikum Wels-Grieskirchen, 4600 Wels, Austria; bjoern.rath@klinikum-wegr.at

**Keywords:** undersampled model, model smoothing, wrist joint, carpal bone

## Abstract

The carpal bones are eight small bones with irregularities and high curvature on their surfaces. The 3D model of the carpal bone serves as the foundation of further clinical applications, e.g., wrist kinematic behavior. However, due to the limitation of the Magnetic Resonance Imaging (MRI) technique, reconstructed carpal bone models are discretely undersampled, which has dramatic stair-step effects and leads to abnormal meshes on edges or surfaces, etc. Our study focuses on determining the viability of various smoothing techniques for a carpal model reconstructed by in vivo gathered MR images. Five algorithms, namely the Laplacian smoothing algorithm, the Laplacian smoothing algorithm with pre-dilation, the scale-dependent Laplacian algorithm, the curvature flow algorithm, and the inverse distance algorithm, were chosen for evaluation. The assessment took into account the Relative Volume Difference and the Hausdorff Distance as well as the surface quality and the preservation of morphological and morphometric properties. For the five algorithms, we analyzed the Relative Volume Difference and the Hausdorff Distance for all eight carpal bones. Among all the algorithms, the scale-dependent Laplacian method processed the best result regarding surface quality and the preservation of morphological and morphometric properties. Based on our extensive examinations, the scale-dependent Laplacian algorithm is suitable for the undersampled carpal bone model with small volume and large curvature.

## 1. Introduction

Wrist pathologies can cause persistent discomfort and motor disorder, which interfere with daily activities and potentially lower life quality. It was reported that around 10% of the general population in the UK suffered nonspecific hand and wrist pain [1]. Another study claimed that wrist and hand pain accounted for a consultation prevalence rate of 190 in 10,000 patients per year in total [2]. Deeper investigation into the wrist mechanism is therefore highly warranted.

Non-invasive interventions are extremely meaningful for either in-vivo or in-vitro investigations into wrists. Medical imaging techniques such as Computerized Tomography (CT) and Magnetic Resonance Imaging (MRI) have been proven to substantially improve diagnosis and treatment procedures for bones [3]. However, most contemporary approaches are confined to 2D representations, which are counterintuitive and sometimes inaccurate regarding spatial information [4]. Compared to 2D images, 3D models provide realistic visualization of anatomical features and can be utilized in orthopedics to visually check the bone shape and joint structure as well as quantitatively assess various clinical states [5], such as osteoarthritis, which may result in the change of bone volume. Mavrogenis et al. demonstrated that employing bone models derived from CT data and computer-assisted navigation systems has been proven to be an effective technique of surgical assistance [6]. A 3D visualization of the wrist bones is of therapeutic importance due to the tiny size and complicated anatomical features of them, which are challenging to observe in 2D images [7]. With 3D carpal models, analyses—e.g., of wrist kinematic behavior—can be directly conducted on a virtual model for therapy planning or surgery assessment [8,9].

**Figure 1:**
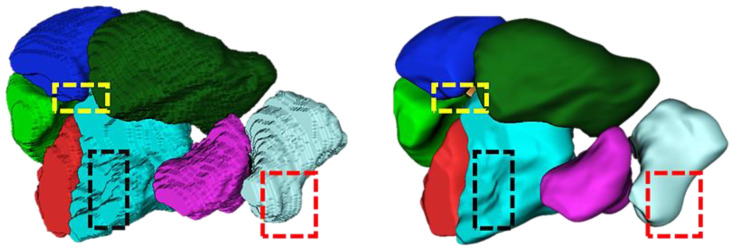
Anatomical information loss. **Left:** Carpal bones shown in the ITK-SNAP in a voxel representation. **Right:** Same bones smoothed by the build-in smoothing function in the ITK-SNAP. Dashed boxes with the same color highlight the anatomical features.

As the precondition of the above-mentioned analysis, the individual bone model plays a fundamental role. The typical process to generate a bone model can be simplified with the following steps: (1) acquisition of stacked image sets; (2) segmentation of the desired objects; (3) reconstruction and smoothing of the object surface. However, due to the small scale (e.g., the volume of the pisiform can be as low as 854 ± 203 mm^3^ in men and 570 ± 122 mm^3^ in women [10]) and large curvature of the carpal bones, the acquisition appears often undersampled. Meanwhile, high-frequency information on stacked 2D images, which may belong to the cortex and sponge of the bones, is rather delicate under image processing. Hence, a smoothing procedure of the bony structures remains necessary. Various algorithms for the surface representation may result in completely different outcomes, e.g., irregular meshes on edges or surfaces with high curvatures. Due to the tiny volume and sophisticated geometry of the carpal bones, volume shrinkage and the ensuing loss of anatomical features during the smoothing procedure degrade the model accuracy massively. The conventional approaches integrated into, e.g., ITK-SNAP [11] or 3DSlicer [12] either simply stack the 2D images with scanning parameters, which results in the stair-step effect, or smooth the model by losing the bony structure dramatically (see Figure 1). By introducing a proper approach, the model surface could be smoothly refined while maintaining essential anatomical features.

Within this paper, we conduct a feasibility study to characterize the smoothing effect of different approaches on the carpal bones, aiming to conclude an optimal approach for carpal bone smoothing. We have made three contributions: (1) we detailed the commonly utilized smoothing algorithms; (2) we implemented the algorithms on our in vivo gathered human wrist MRI sets; (3) we then compared and explained the results mathematically of each algorithm and offered a fitted smoothing strategy for carpal bones with high quality and accuracy.

## 2. Materials and Methods

### 2.1. Data Acquisition and Preprocessing

The datasets involved in this study were adapted from our former research, which has been approved by the institutional review board (EK 171/10), and written informed consent requirements were obtained [13]. We randomly selected MRI scans and corresponding segmented ground truths from ten subjects. The reconstructed models were directly outputted through the ITK-SNAP without any further build-in smoothing procedures.

### 2.2. Laplacian Smoothing

Laplacian smoothing is a typical approach for polygonal mesh smoothing, which is also known as diffusion. The essential principle is that the vertices of a mesh are shifted in the Laplacian direction in steps. The smoothing equation is given in differential form as follows:

(1) $$\frac{\partial X}{\partial t}=\lambda L\left(X\right),$$

where X denotes the mesh vertices, L is the Laplacian operator, and λ is a positive coefficient that controls the diffusion speed.

**Figure 2:**
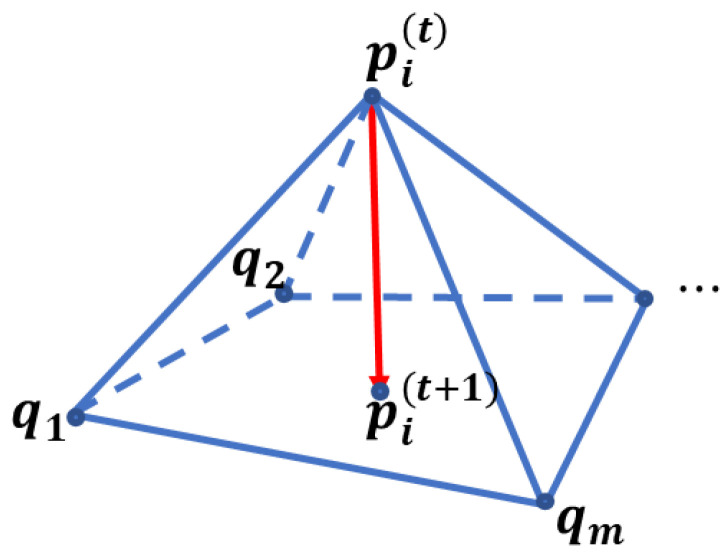
The umbrella operator approximating the Laplacian smoothing on a mesh. $q_{1}$ to $q_{m}$ are the points on the one-ring neighbor of the vertex $p_{i}$.

More rigorously, consider that a mesh M with vertex set V={1,…, n} is given by a tuple (K,p), where $K\subseteq 2^{V}$ is a simplicial complex and $p:V\to {\mathbb{R}}^{3}$ maps the vertex i∈n to its position $p_{i}$. Laplacian smoothing can be discretely approximated as a repeated replacement of position $p_{i}$ of the vertex i by the umbrella operator $U\left(p_{i}\right)$ [14,15] to the following:

(2) $$p_{i}^{\left(t+1\right)}=p_{i}^{\left(t\right)}+\lambda U{\left(p_{i}\right)}^{\left(t\right)},$$

(3) $$U\left(p_{i}\right)=\frac{1}{m}\left(\underset{j\in N_{1}\left(i\right)}{\sum }q_{j}\right)-p_{i},$$

where $N_{1}\left(i\right)$ denotes the one-ring neighbors of the vertex i and m is the number of neighbors of the $p_{i}$ (see Figure 2). The umbrella operator U can be seen as a low-pass filter.

### 2.3. Laplacian Smoothing with Pre-Dilation

As mentioned previously, Laplacian smoothing reduces the high-frequency signals and hence causes volume shrinkage. To avoid this volume loss, we propose a pre-dilation algorithm before the smoothing to compensate for such an impact.

Given a position $p_{i}=\left(x_{i},y_{i},z_{i}\right)$ of the vertex i of a mesh M, the dilation is defined as follows:

(4) $$\{\begin{array}{c} x_{i}=x_{i}+\alpha \cdot n_{x_{i}}\\ y_{i}=y_{i}+\alpha \cdot n_{y_{i}}\\ z_{i}=z_{i}+\alpha \cdot n_{z_{i}} \end{array}\;,$$

where α is a positive coefficient and $n_{x}$, $n_{y}$, and $n_{z}$ represent the normal components of the corresponding point. Since the total complexity for volume preservation is linear, the dilatation is frequency-insensitive. When compared to previous scaling methods, our technique can maintain the absolute location.

The model volume reduces gradually throughout the Laplacian smoothing, and the process is terminated as (1) the smoothed surface contacts the boundary of the original surface or (2) the volume difference between the smoothed model and the original model reaches a certain threshold (e.g., 0.5% of the original volume). The Algorithm 1 is summarized underneath.
**Algorithm 1:** Laplacian smoothing with pre-dilation**Input:**$V^{t}$, $p^{t}$, λ, α, threshold**Output:**$V^{t+1}$, $p^{t+1}$1.**Initialization**: λ**,** α2.**Dilation** using Equation (4)3.**while**volume difference**>**threshold **do**4.  
$p_{i}^{\left(t+1\right)}⟵\;p_{i}^{\left(t\right)}+\lambda U{\left(p_{i}\right)}^{\left(t\right)}$
5.  volume difference
**=** volume of $V^{t+1}$
**−** volume of $V^{t}$6.**end while**

### 2.4. Scale-Dependent Laplacian Smoothing

Typically, we utilize the umbrella operator to approximate the Laplacian operator. As expressed in Equation (2), the vertex is assumed to have edges with the same length, and all angles between neighboring edges around the vertex are equal. Since actual meshes have a variety of triangles of varying sizes, the scale-dependent umbrella operator is defined as follows [16,17]:

(5) $$U\left(p_{i}\right)=\frac{2}{E}\underset{j\in N_{1}\left(i\right)}{\sum }\frac{p_{j}-p_{i}}{\left|e_{ij}\right|},$$

(6) $$E=\underset{j\in N_{1}\left(i\right)}{\sum }\left|e_{ij}\right|,$$

**Figure 3:**
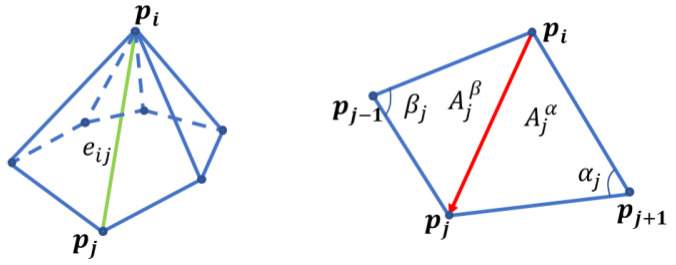
Calculation of the mean curvature around a vertex. $e_{ij}$ is the edge connecting $p_{i}$ to $p_{j}$.

As illustrated on the left side of Figure 3, $e_{ij}$ is the edge connecting the vertices in positions $p_{i}$ and $p_{j}$.

### 2.5. Curvature Flow

The smoothing approach based on curvature flow makes no use of the surface’s inherent features. It smooths the surface by moving along the surface normal n with a speed equal to the mean curvature $\bar{\kappa }$ [18]. Equation (1) can be rewritten as follows:

(7) $$\frac{\partial x_{i}}{\partial t}=-\bar{\kappa_{i}}n_{i},$$

(8) $$\bar{\kappa_{i}}=\frac{\kappa_{1}+\kappa_{2}}{2}.$$

The definition of the mean curvature around a vertex is defined as follows:

(9) $$\bar{\kappa }=\mathrm{div}\;n,$$

where div(⋅) is a vector operator in vector analysis that corresponds a vector field in a vector space to a scalar field [19].

We can use a differential geometry curvature as an approximation to the (x,y,z) coordinates of the vertex to the following:

(10) $$\bar{\kappa }n=\frac{\nabla A}{2A},$$

where A is the area of a small region around the vertex and ∇ denotes the gradient. A vertex $x_{j}$ in its position $p_{j}$ and the area of all the triangles of the one-ring neighbors would be taken into consideration, while the area of the triangle uses the cross-products of adjacent edges.

The expression is reformed as follows:

(11) $$-\bar{\kappa }n=\frac{1}{4A}\underset{j\in N_{1}\left(i\right)}{\sum }\left(\cot\alpha_{j}+\cot\beta_{j}\right)\left(p_{j}-p_{i}\right).$$

In the equation above, $\alpha_{j}$ and $\beta_{j}$ are two angles opposite to the edge in the two triangles having the edge $e_{ij}$, and $A_{j}^{\alpha }$ and $A_{j}^{\beta }$ are the areas of the triangles, as shown in Figure 3.

Similar to previous approaches, we may reduce the non-linear formulation of the curvature normal method into the following linear system with an implicit integration known as the backward Euler method [17]:

(12) $$\left(I-\lambda \;dt\;K\right)\;P^{n+1}=P^{n}.$$

Then, the equation can be normalized to be used in explicit integration for quick smoothing [17]:

(13) $${\left(\bar{\kappa }n\right)}_{normalized}=\frac{1}{\Sigma_{j}\left(\cot\alpha_{j}^{l}+\cot\alpha_{j}^{r}\right)}\underset{j}{\sum }\left(\cot\alpha_{j}^{l}+\cot\alpha_{j}^{r}\right)\left(P_{i}-P_{j}\right).$$

### 2.6. Inverse Distance

From Equations (3)–(5), it is obvious that the definition of the weights $\omega_{i}$ in the umbrella operator varies the smoothing strategy, e.g., $\omega_{i}=\frac{1}{m}$ in Equation (3).

Another option of the weights is the inverse distances between P and its neighbors $Q_{i}$ [20], as shown in Figure 2:

(14) $$\omega_{i}=\|P-Q_{i}\|{}^{-1}.$$

The local update rule still follows Equation (2).

### 2.7. Validation

Since the real carpal bone surface geometry cannot be completely obtained in vivo and any smoothing process on the model surface causes unavoidable loss of anatomical information, we selected models directly outputted by the ITK-SNAP without any built-in processing as ground truths, where every convex ridge represents a real anatomical boundary on the selected carpal bone, and then compared them to smoothing results from the employed approaches to characterize the approach that offered the best compromise between smoothness and fidelity.

Therefore, we utilized various methods for the validation of the referred smoothing methods, which are described underneath. For convenience, we denote the Laplacian smoothing method as M1, the Laplacian method with pre-dilation as M2, the scale-dependent Laplacian method as M3, the curvature flow method as M4, and the inverse distance method as M5. We empirically applied a 5% dilatation on the origin model as input for M2. As M2 converges automatically, all other methods were manually set to terminate after 30 iterations.

#### 2.7.1. Quantitative Metrics

We employed the Relative Volume Difference (RVD, %) and the Hausdorff Distance (HD, mm) as the surface distance measures. The VD and the HD are defined as follows:

(15) $$\mathrm{RVD}\left(G,S\right)=\frac{\left|V_{S}\right|-\left|V_{G}\right|}{\left|V_{G}\right|}\cdot 100\;,$$

(16) $$\mathrm{HD}\left(G,S\right)=\max\left(\underset{x\in G}{\max}d\left(x,S\right),\underset{y\in S}{\max}d\left(x,G\right)\right).$$

where G and S represent ground truth and segmentation, respectively; d(a,b) the nearest Euclidean distance between a surface point a and the surface b; and |·| the number of corresponding surface voxels. A value of the RVD and the HD equal to zero means a perfect segmentation.

As the ridges on the ground truth model portray the real anatomical geometry, the HD implies how the involved approach maintains such information by measuring the maximum distance between two comparisons. The RVD measures the degree of the volume change. Since the RVD is given as a signed number, it reveals model expansion or shrinkage as well.

#### 2.7.2. Qualitative Metrics

Following quantitative assessment, we further investigated how the employed smoothing approaches preserved the anatomical features, which can intuitively identify the discrepancies in smoothing outcomes. Specifically, we compared the morphological and morphometric features of the scaphoid and trapezium due to their highly complex spatial geometries [21,22].

**Figure 4:**
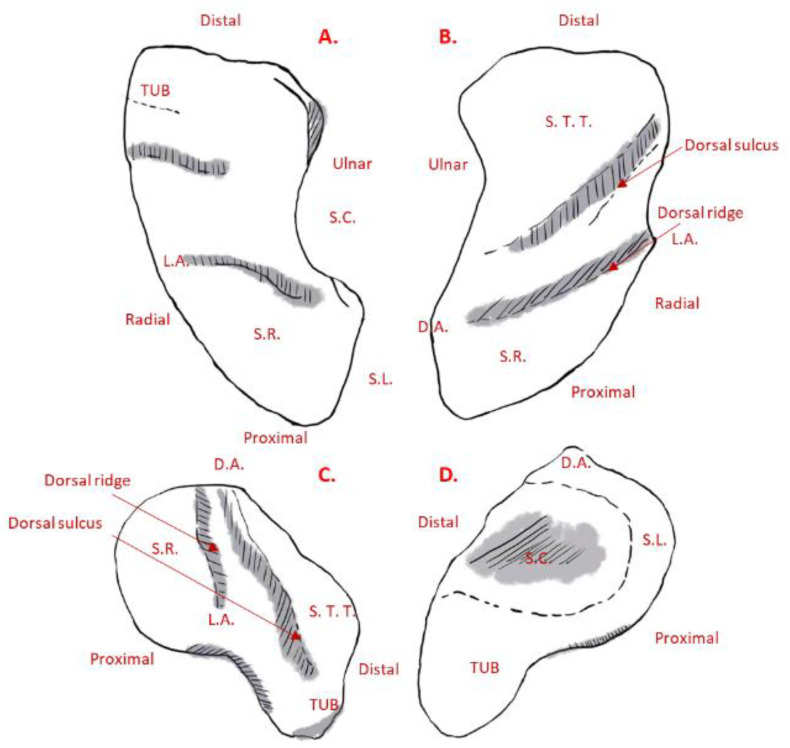
(**A**) Palmar, (**B**) dorsal, (**C**) radial, and (**D**) ulnar views of a left scaphoid. *D.A.* = Dorsal apex of the ridge. *L.A.* = Lateral apex of the ridge. *TUB* = Tubercle. *S.C.* = Scapho-capitate joint. *S.L.* = Scapho-lunate joint. *S.R.* = Scapho-radial joint. *S.T.T.* = Scapho-trapezium-trapezoid joint.

Compson et al. outlined the morphological landmarks of the scaphoid as follows [21]: (1) the scaphoid has six facets, four of which are articular facets; (2) the lunate facet is semilunar in shape and faces medially and slightly into the palmar direction; (3) the capitate facet is larger and concave and faces medially and slightly distally; (4) the dorsal edge of the capitate facet is more concave than the palmar and can contain a notch; and (5) the position of the capitate facet varies in its relationship to the proximal end of the scaphoid, which leads to an associated variability in the width of the lunate facet (see Figure 4).

**Figure 5:**
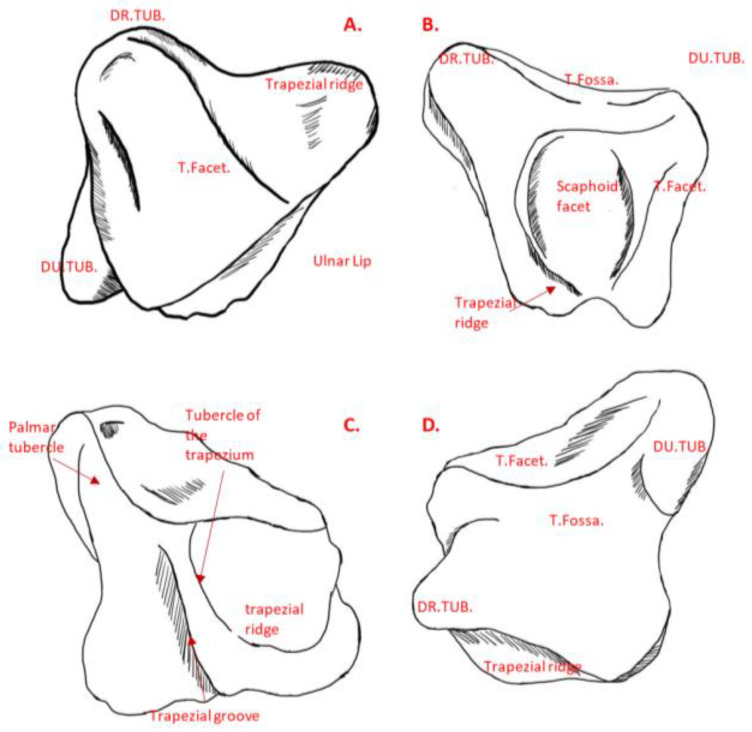
(**A**) Thumb metacarpal, (**B**) proximal, (**C**) palmar, and (**D**) dorsal views of a trapezium. *DR.TUB.* = Dorso-radial tubercle. *T.Facet.* = Thumb metacarpal facet. *T.Fossa.* = Thumb metacarpal fossa. *DU.TUB.* = Dorso-ulnar tubercle.

The morphological landmarks of the trapezium are as follows [23]: (1) the trapezium displays six facets, comprising four articular facets and two other non-articular palmar and dorsal facets; (2) the largest articular facet is the thumb metacarpal facet, which is saddle-shaped and faces distally and radially (see Figure 5D), being radioulnarly concave while palmardorsally convex; (3) the second largest of the articular facets is the trapezoid, which is elongated and concave and faces ulnarly as the most irregular surface (see Figure 5C); (4) the concave scaphoid facet articulates with the tubercle of the scaphoid, and it is rounded and the most proximal of all the articular surfaces; (5) the most prominent feature on the palmar facets is the trapezial ridge (see Figure 5C), which is a bony ridge that runs from proximal to distal, facing ulnarly; and (6) the dorsal facets displays two distinct tubercles (see Figure 5D).

## 3. Results

### 3.1. Quantitative Metrics

As listed in Table 1, M1 suppressed the volume the most. Compared to M1, M2 performed exceptionally well in terms of volume retention. M3, M4, and M5 all had a comparable effect on mitigating the volume shrinking effect during the smoothing process.

The mean HD and Root Mean Square (RMS) of the five methods on eight carpal bones are shown in Table 2. The HDs of M3, M4, and M5 have similar standard deviations and are all smaller than those of M1 and M2.

**Figure 6:**
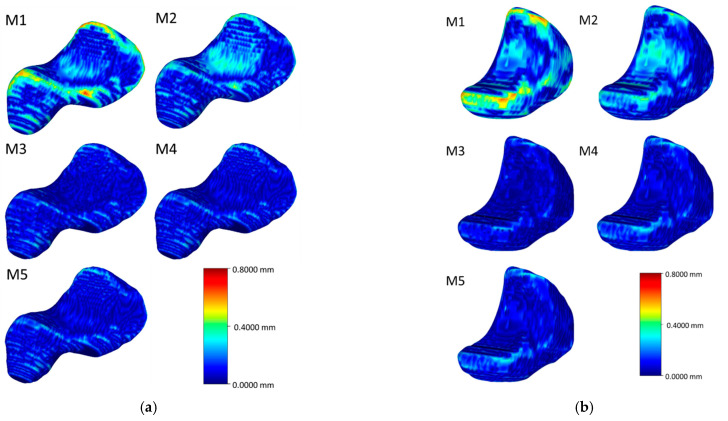
Heat map of the HD between the original model and the model after processing of the scaphoid (**a**) and the lunate (**b**).

**Figure 7:**
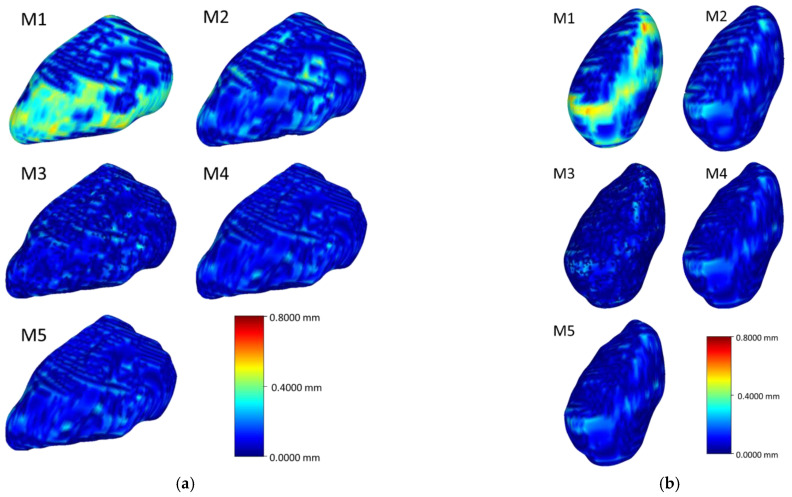
Heat map of the HD between the original model and the model after processing of the triquetrum (**a**) and the pisiform (**b**).

**Figure 8:**
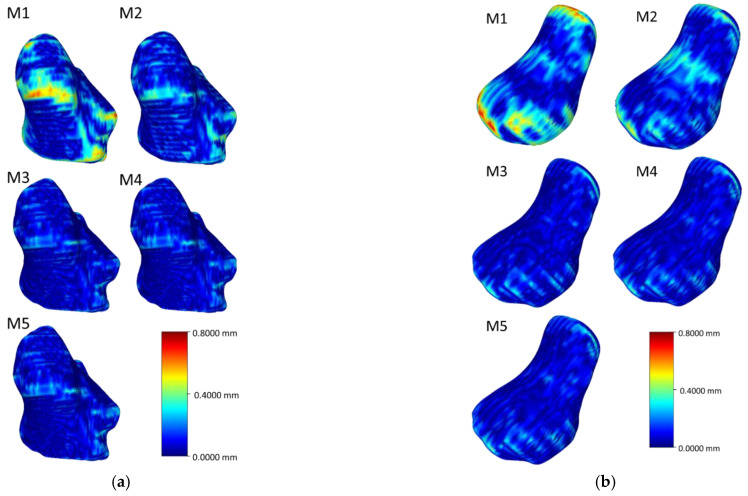
Heat map of the HD between the original model and the model after processing of the trapezium (**a**) and the trapezoid (**b**).

**Figure 9:**
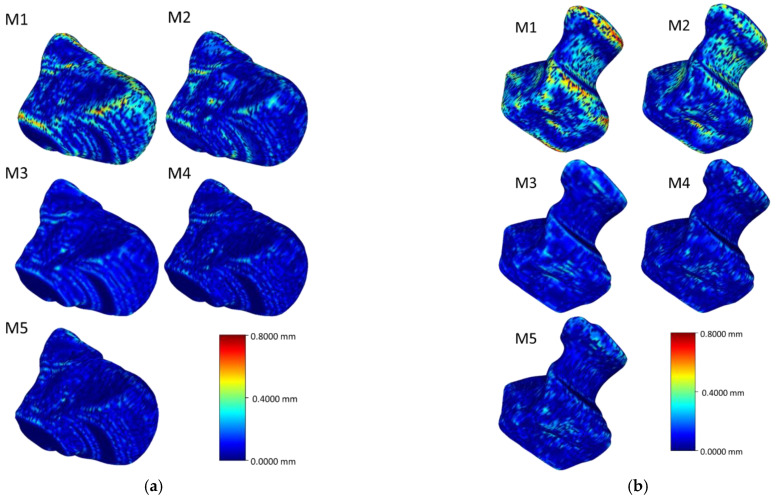
Heat map of the HD between the original model and the model after processing of the capitate (**a**) and the hamate (**b**).

Figure 6, Figure 7, Figure 8 and Figure 9 visualize the HD distribution along the bone surface smoothed by the approaches employed on all of the eight carpal bones. Generally, M1 has the largest HD distribution and M3 has the smallest on all the bones. M2 performs better than M1; however, M2 does not work well at the surface with negative curvature (the scaphocapitate facet in Figure 6a). M3, M4, and M5 share a similar outcome, although M3 achieves less distortion on the edge (shown in Figure 6b).

### 3.2. Surface Quality

**Figure 10:**
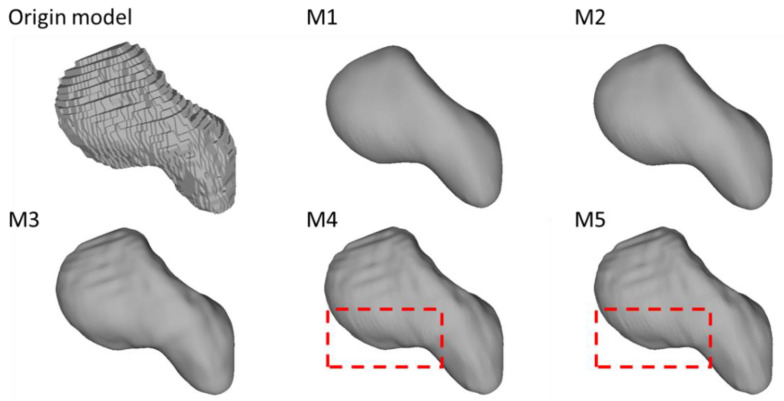
Smoothing quality of models smoothed by different approaches. *Red dashed boxes:* Oscillatory ripples on the surface.

The surface quality for the used algorithms is shown in the following section. Figure 10 demonstrates the original model of a scaphoid bone and the results of M1 to M5.

The original model suffers from a stepwise surface due to the directly stacked segmentation slices. All the smoothing methods significantly improved the surface quality by reducing the stair-step effect in the original model. M1 and M2 offered the best result regarding surface smoothness. M3, M4, and M5 share similar results, where the majority of the stepped edges were eliminated while some of them remained. Additionally, compared to M3, M4 and M5 generated unnecessary local oscillatory ripples on the edges.

### 3.3. The Preservation of the Morphological and Morphometric Features

**Figure 11:**
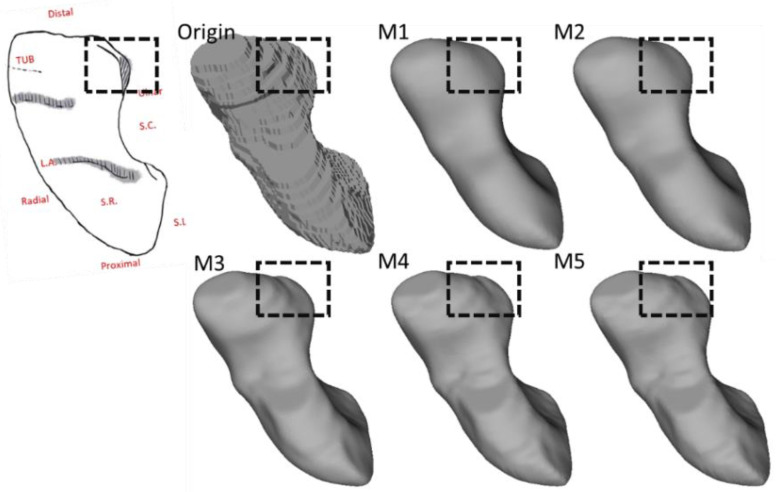
The preservation of the morphological features on the distal side of the scaphoid. All models are presented in the same orientation. *Black dashed boxes:* Loss of anatomical features.

**Figure 12:**
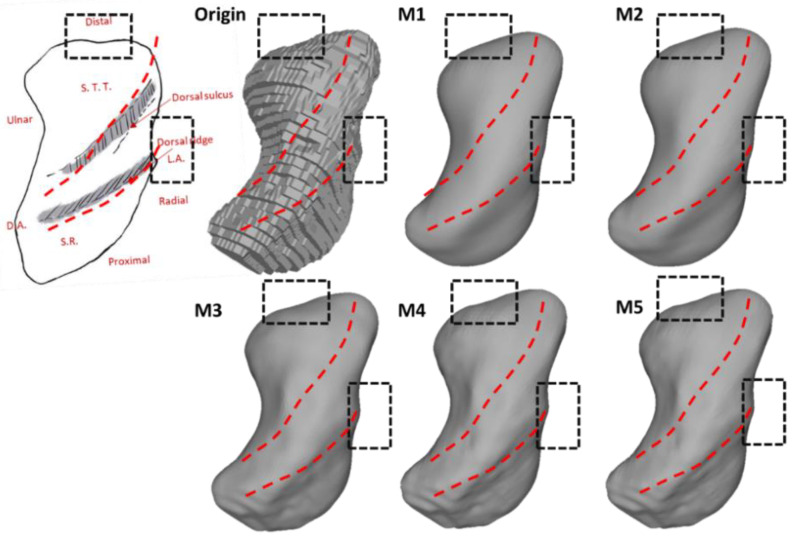
The preservation of the dorsal sulcus and dorsal ridge of the scaphoid. All models are presented in the same orientation. *Black dashed boxes:* Loss of features. *Red dashed lines:* Features on the bone surface.

**Figure 13:**
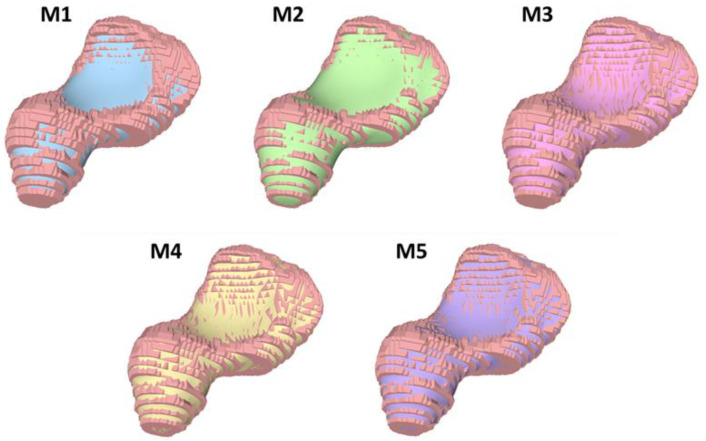
Comparison of scaphoid model overlap. *Pink:* Original model obtained by segmentation; *Blue:* M1; *Green:* M2; *Plum:* M3; *Yellow:* M4; *Purple:* M5.

Figure 11 and Figure 12 illustrate the morphological characteristics of the scaphoid and their preservation status. The comparison between M1, M2, M3, M4, and M5 clearly illustrates the damage of the different smoothing strategies regarding morphological and morphometric features. As shown in those figures, both M1 and M2 have a considerable negative effect regarding the form of the models, resulting in a significant deterioration of the anatomical features. In Figure 13, we also notice the unnecessary expansion on the scaphocapitate concave facet.

**Figure 14:**
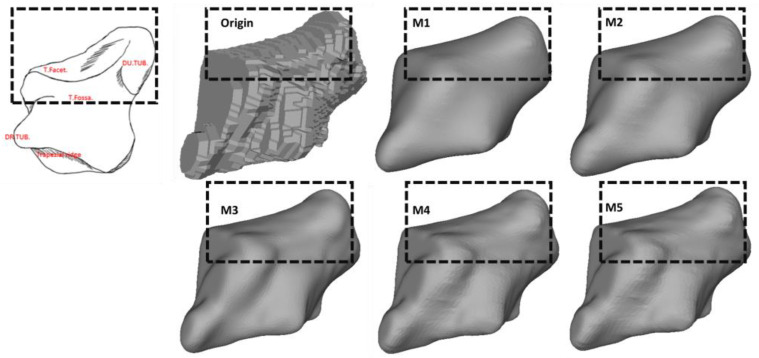
The preservation of the thumb metacarpal facet of the trapezium. All models are presented in the same orientation. *Black dashed boxes:* Loss of features.

**Figure 15:**
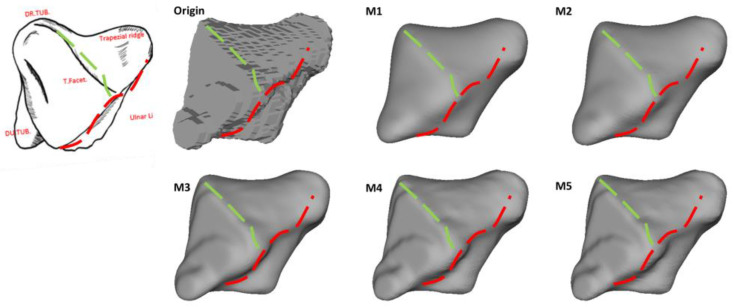
The preservation of the trapezial ridge of the trapezium. All models are presented in the same orientation. *Red and green dashed lines:* Loss of features.

The preservation of the morphological characteristics of the trapezium is displayed in Figure 14 and Figure 15. As a result of the smaller bone volume compared to the scaphoid, the morphological features on the trapezium were generally preserved. In Figure 14, unlike the results from M3–M5, both M1 and M2 have a remarkable negative effect on the preservation of the thumb metacarpal facet. Figure 15 shows the preservation of the trapezial ridge. All approaches preserve the trapezial ridge, though the results in M3–M5 are better compared to M1 and M2.

## 4. Discussion

While M1 effectively improves the model’s surface quality, according to Equations (2) and (3), M1 suppresses vertices on the surface at each iteration, resulting in a significant volume reduction, which is incompatible with objects with limited volume and considerable curvatures such as the carpal bones. Meanwhile, since each step on the original model represents a real bony contour, M1 loses anatomical features the most.

Such volume shrinkage can be relieved by implementing dilatation before Laplacian operation, as in M2. Nevertheless, the expansion of M2 is rather empirical, which is individual-dependent and thus hardly practical for routine processing. Furthermore, the processing might damage the crucial surface morphological and morphometric properties as M2 shares the same smoothing strategy as M1. Figure 13 shows that the model processed by M2 expands on the scaphocapitate facet, and the articular concavity moves slightly centripetally, which decreases the scaphocapitate distance, resulting in a potential interference on inter-bone kinematic characters or even a cartilage modeling failure in terms of a more detailed wrist model. Such a drawback is explained through Equation (4), which enlarges the model homogeneously by setting the normal direction of individual voxels outwards generally. As a result, the sections of the model surface with positive curvature and negative curvature are equally unduly extended. Thus, the scaphocapitate facet curvature varies when no compensation through the following Laplacian operation is performed.

M3, M4, and M5 smooth the models in a similar manner, as all of them are variations of the traditional Laplacian method. M4 and M5 perform admirably in terms of volume preservation and morphological and morphometric feature retention as they do not change the model shape at the facet that is flat. Therefore, fewer vertices are to be handled, leading to fewer differences between the smoothed and original models and a smaller HD listed in Table 2. However, in Figure 6, the methods do not converge at locations with small curvature during the smoothing, which causes oscillatory ripples on the surface. It is reported that according to Equations (13) and (14), the nonlinear evolutions of M4 and M5 under an unstable flow or in a finite time interval exhibit unstable behavior [24,25].

M3 offers the premier smoothing results regarding the carpal bones among all the comparisons. As expressed in Equation (6), by adapting the edges of the umbrella operator depending on each vertex, M3 avoids an indistinguishable high-frequency filtration along the surface, which limits the volume shrinkage compared to M1. Although the length of the edges drives the operator to become nonlinear, it varies within a certain level. Desbrun et al. reported that the operator of M3 remains constant during numerical implementation [17] and could therefore avoid oscillation.

Indeed, we noticed the limitations of this work. Due to the principle of MRI, the discrete acquisition offers limited anatomical features of carpal bones. The segmentations and the resultant generated ground truth models can hardly completely mimic the real scenario. Meanwhile, based on the project demands, we mainly focused on the implicit approaches derived from Laplacian smoothing. An explicit Euler scheme-based approach or other embedded kernel function-based approaches, such as Gaussian kernel, remain promising research topics. Furthermore, the application can also be extended to other joints, e.g., tarsus or vertebra, which have similar surface features.

## 5. Conclusions

In this work, the performance of various smoothing methods on the carpal bones has been investigated for the first time. We pointed out the best solution for smoothing the medical objects with large curvature and undersampled by MRI. Specifically, we first expressed five commonly employed smoothing methods mathematically, i.e., Laplacian smoothing (M1), Laplacian smoothing with pre-dilation (M2), scale-dependent Laplacian smoothing (M3), curvature flowing smoothing (M4), and inverse distance smoothing (M5). We then implemented the methods on our in-house gathered in vivo MR carpus image sets. The results were quantitatively—i.e., through Relative Volume Difference and Hausdorff Distance—and qualitatively—i.e., through morphological and morphometric features—evaluated. We then discussed the outcomes and the main drawbacks of the methods mathematically.

Generally, Laplacian smoothing is appropriate when the model processes a considerable volume, and the assessment criterion is loose regarding local feature preservation. The dilation algorithm can be utilized in conjunction with the Laplacian smoothing algorithm to achieve the desired volume preservation. The curvature flowing smoothing and inverse distance smoothing methods showed a great performance to shape. However, optimization is still needed to solve the problem of surface oscillation.

We recommend employing scale-dependent Laplacian smoothing for large curvature, small volume, and undersampled models since it can optimize the surface quality to an acceptable level while maintaining the model volume and the anatomical details demanded.

## Figures and Tables

**Table 1:** RVD (in %) of five methods on eight carpal bones.

Method	Scaphoid	Lunate	Triquetrum	Pisiform	Trapezium	Trapezoid	Capitate	Hamate
M1	−7.139	−7.625	−9.1	−12.59	−7.292	−8.755	−5.589	−6.379
M2	0.3133	0.436	0.181	0.3328	0.4872	0.4425	0.3548	0.3525
M3	−1.526	−1.667	−1.925	−2.723	−1.653	−2.217	−1.114	−1.453
M4	−1.373	−1.479	−1.755	−2.414	−1.456	−1.82	−1.043	−1.298
M5	−1.445	−1.554	−1.842	−2.527	−0.01525	−0.01883	−0.01097	−0.01361

**Table 2:** HD (in mm) of five methods on eight carpal bones.

Method	Scaphoid	Lunate	Triquetrum	Pisiform	Trapezium	Trapezoid	Capitate	Hamate
M1	0.176 ± 0.217	0.188 ± 0.226	0.198 ± 0.242	0.205 ± 0.244	0.180 ± 0.227	0.193 ± 0.241	0.170 ± 0.214	0.199 ± 0.245
M2	0.118 ± 0.145	0.115 ± 0.144	0.121 ± 0.1472	0.096 ± 0.117	0.121 ± 0.151	0.129 ± 0.160	0.124 ± 0.153	0.146 ± 0.182
M3	0.075 ± 0.096	0.074 ± 0.095	0.079 ± 0.101	0.083 ± 0.102	0.082 ± 0.104	0.079 ± 0.103	0.073 ± 0.096	0.075 ± 0.098
M4	0.073 ± 0.093	0.072 ± 0.093	0.076 ± 0.097	0.077 ± 0.099	0.079 ± 0.101	0.077 ± 0.100	0.070 ± 0.093	0.075 ± 0.098
M5	0.074 ± 0.094	0.074 ± 0.095	0.077 ± 0.098	0.081 ± 0.099	0.081 ± 0.103	0.079 ± 0.102	0.072 ± 0.095	0.077 ± 0.101

## Data Availability

Not applicable.
